# Plaster of Paris vs. Modeling Composition

**Published:** 1883-03

**Authors:** Stewart J. Spence


### ARTICLE VII.

### *Plaster of Paris vs. Modeling Composition.*

BY DR. STEWART J. SPENCE.

In the *Ohio State Journal of Dental Science* of November last Dr. U. Smith had an article "About Impressions," condemning plaster and recommending Modeling Composition No. 1. His claim is, that the composition, by compressing the softer tissues of the mouth, provides for a plate which will do similarly, and therefore be more permanently useful.

That provision should be made for this compression of the soft parts by a plate, is of the greatest importance. Harris recognizes it, and recommends, if I recollect right, brushing thin plaster over the impression at the parts corresponding to the soft parts of the mouth.

Richardson dwells upon the subject, and points out that "the tenacity with which a plate adheres, on the application of direct traction, cannot always be relied upon, inasmuch as a well fitting plate will sometimes be readily dislodged in this manner, while, on the contrary, one but illy adapted to the parts, may require considerable force to separate it from the jaw, when acted on in the same way. The most trustworthy test of actual, or practical stability, is firm pressure, applied alternately over the ridge on each side, and in front."—*Mechanical Dentistry*, p 252.

Several other writers enforce the subject, some recommending treating the impression with a coating of varnish at the soft parts, or a few layers of tin foil, and others trimming down the model with a knife.

Harris says: "A vulcanite plate is larger than the mouth by the expansion of the model. Here, the contraction of gutta percha will prove a very valuable compensation, *also the compression of tissue, made by the pressure of wax.*"—*Principles and Practice of Dentistry*, p 548.

I dwell on this subject because of its importance, and I am glad to see it brought forward by Dr. Smith; but I must disagree with him as to the excellency of the Godiva Modeling Composition, and for these reasons; *It is elastic and pliable.* Though very pleasant to use, clean, and easy of application, it is treacherous. While it is very accommodating in taking the faintest depressions so as to delude the eye of the operator, it will bend out of shape in a wholesale manner. While wax, which has little elasticity, will, in being removed from a tooth, drag just at the tooth, locally, so to speak, the composition will drag not so much just at the tooth as around it, and that for some distance. I have noticed it thus dragged by a tooth away from the tray in lower impressions, thus throwing up that whole side of the impression, Nor is that all, but its elasticity developes itself in another feature, which is, that it has a tendency not to remain where placed. This is most troublesome at the buccal and labial surfaces of the upper jaw, where it becomes necessary to hold the material pressed until quite hard, or it will shrink away. This may be demonstrated by pressing a plaster model into a tray full of the Godiva, when it will be seen that the composition which is above the rim of the tray will shrink from the plaster, *and when pressed up against it will shrink away again as soon as the pressure is removed.* I am not prepared to deny that the pressure of the lips and cheeks may, in some cases, more or less counteract this shrinking away but in others it seems to increase it.

Again, its specific gravity being considerable, and its resistance very slight, it bends from its own weight, when used very warm and soft, thus coming away from the parts where it may have been pressed, and possibly from the posterior portion of the plate, though I am not able to speak positively as to this. A sufficient excess of the material may prevent it falling from the surfaces which are to be covered by the plate; and unless used *too* soft, I think it is not liable to occur. The same objection applies to the plaster if used too soft.

This pliability would not be an objection with the inferior ridge, and might be some benefit. But when used very soft, it is deprived of the quality for which Dr. Smith recommends it, namely: compression of soft tissue; and the doctor seems to have overlooked the fact that plaster, when used stiff, will do this very thing.

Not condemning this nice material *in toto*, I desire to only indicate its faults, so that those using it may be on their guard; and I would recommend constant digital compression where needed, high flangs to trays, its use when not too warm and soft, and replacing the impression in the mouth after removing and immersing it in cold water. —*Ohio State Journal of Dental Sciencc.*

